# Perspectives on modelling the distribution of ticks for large areas: so far so good?

**DOI:** 10.1186/s13071-016-1474-9

**Published:** 2016-03-31

**Authors:** Agustín Estrada-Peña, Neil Alexander, G.R. William Wint

**Affiliations:** Department of Animal Pathology, Faculty of Veterinary Medicine, Miguel Servet 177, 50013 Zaragoza, Spain; Environmental Research Group Oxford, Department of Zoology, South Parks Road, Oxford, OX1 3PS UK

**Keywords:** Correlative distribution modelling, Ticks, Remote sensing, MODIS Fourier transformation

## Abstract

**Background:**

This paper aims to illustrate the steps needed to produce reliable correlative modelling for arthropod vectors, when process-driven models are unavailable. We use ticks as examples because of the (re)emerging interest in the pathogens they transmit. We argue that many scientific publications on the topic focus on: (i) the use of explanatory variables that do not adequately describe tick habitats; (ii) the automatic removal of variables causing internal (statistical) problems in the models without considering their ecological significance; and (iii) spatial pattern matching rather than niche mapping, therefore losing information that could be used in projections.

**Methods:**

We focus on extracting information derived from modelling the environmental niche of ticks, as opposed to pattern matching exercises, as a first step in the process of identifying the ecological determinants of tick distributions. We perform models on widely reported species of ticks in Western Palaearctic to derive a set of covariates, describing the climate niche, reconstructing a Fourier transformation of remotely-sensed information.

**Results:**

We demonstrate the importance of assembling ecological information that drives the distribution of ticks before undertaking any mapping exercise, from which this kind of information is lost. We also show how customised covariates are more relevant to tick ecology than the widely used set of “Bioclimatic Indicators” (“Biovars”) derived from interpolated datasets, and provide programming scripts to easily calculate them. We demonstrate that standard pre-tailored vegetation categories also fail to describe tick habitats and are best used to describe absence rather than presence of ticks, but could be used in conjunction with the climate based suitability models.

**Conclusions:**

We stress the better performance of climatic covariates obtained from remotely sensed information as opposed to interpolated explanatory variables derived from ground measurements which are flawed with internal issues affecting modelling performance. Extracting ecological conclusions from modelling projections is necessary to gain information about the variables driving the distribution of arthropod vectors. Mapping exercises should be a secondary aim in the study of the distribution of health threatening arthropods.

**Electronic supplementary material:**

The online version of this article (doi:10.1186/s13071-016-1474-9) contains supplementary material, which is available to authorized users.

## Background

Environmental suitability varies within the range of virtually all species, causing patchy rather than homogeneous distributions, with a presumed lower abundance in the less suitable areas [1]. It is this environmentally driven heterogeneity that underpins species distribution modelling (i.e. habitat modelling, ecological, environmental, or climate niche modelling), which all identify places suitable for the survival of populations by identifying their environmental requirements [2]. These models commonly use associations between environmental variables and known species occurrence and/or absence records to identify conditions within which populations can be maintained [3, 4].

The niche concept was popularized in 1957 by Hutchinson [5]. The Hutchinsonian niche is an n-dimensional hypervolume, where the dimensions are environmental conditions and resources that define the requirements for a population to persist. The niche can thus be mathematically quantified and the “position” of a species in the n-dimensional volume plotted and analysed. An “environmental niche” can be defined as the combination of a series of (often climatic or vegetation related) variables that influence survival and reproductive rates, thereby “driving” the population growth [6] and limiting a species’ range. A broader definition of the niche can incorporate features of topographic parameters (like the slope or aspect), as well as demographic, social and agricultural features of an area. In the case of parasitic arthropod vectors, “biotic” features of their life - cycles, like the availability, number and abundance of host species used, can also play an important role in sustaining or restricting population growth in addition to the climate conditions [7].

There is an increasing interest in predicting the possible effects of changing climate on the distributions of parasitic arthropods. Studies have been carried out on many organisms, including mosquito, sandfly or tick vectors [i.e. 8–10] which capture, at various spatial resolutions, the factors driving their distribution, and attempt to understand how variation in these factors might shape future distributions. The (often mapped) results of these modelling exercises are used to disseminate information and help decision makers produce strategies of preparedness, adaptation or response. We acknowledge that the best methods for modelling the impact of environmental variables on the tick life - cycle are process-driven models because they describe each development or mortality process [11]. The lack of knowledge of the drivers of physiological processes for many species of ticks precludes their use on a wide scale, and correlative or statistical distribution modelling is therefore extensively used instead. The validity of these methods is sometimes, however, undermined by several methodological issues which can arise from an insufficient understanding of a) the biology of the organism to be modelled, b) the statistical rules that drive the concept of niche modelling, or c) which variables should be used to build the models. For example: an association with a covariate does not prove cause and effect [12] and so extrapolating such associations to future climate scenarios may not be appropriate as our current knowledge of ticks is often insufficient to reliably predict the impact of changing environments on tick biology. Instead, the scientific landscape of tick modelling has focused on relatively few methods, and has very often relied on a single set of explanatory variables derived from interpolated climatic datasets [13], which have not always proven suitable for correlative modelling of tick distributions [14].

This paper explores the process of correlative tick distribution modelling based on a large and updated dataset of tick records from the Palaearctic region [10] which are made available in the supplementary information of this paper. We emphasize that this paper is neither a review of statistical methods, nor a comparison of the performance of different techniques. Our aim is rather to use the results of *ad hoc* analyses, in the context of recently published developments, to show how best to implement the models, focussing on several discrete stages. The first is producing the habitat suitability models including: (i) choosing raw covariates; (ii) producing derivatives that are relevant to tick ecology and incorporating them into models of tick suitability; (iii) mapping the outputs in a way that allows flexibility of presentation. The second stage illustrates how to use the suitability models to identify which of several types of environmental characteristic are associated with high suitability, including; (i) combinations of the predictor covariates; (ii) additional customised ecologically meaningful derivatives of covariates; and (iii) land use/land cover.

In selected cases, we provide scripts written in the widely used R programming environment [15] to improve the understanding of the methods explained here and to enable readers to do their own analyses. We will not explicitly address the issues of the presentation of results, which are usually delivered in the form of maps, but will provide arguments to use a plot of the niche in the environmental rather than spatial dimensions, arguing the importance of defining the drivers of tick distributions, something rarely explored in this field of research.

### The choice between interpolated or remotely sensed environmental covariates

Correlative spatial modelling aims to establish how environmental covariates (or predictors) are associated with distribution patterns. This section focusses on the use of eco-climatic variables and deliberately ignores the other possible drivers of distribution, such as host presence, socio-economic factors, transport, trade in animals, or geographic barriers that may affect the spread of ticks [16].

There is a growing tendency to use interpolated weather station datasets [13] to identify the geographical range in which arthropod vectors may survive and then to project these trained models into future scenarios. These data are enormously useful to describe large scale patterns in climate, if the stations are close together, but if they are far apart, as in many remote areas, interpolations are increasingly inaccurate. In addition, because they are interpolated, monthly summaries suffer from significant co-linearity and auto-correlation [14, 17, 18]. As a result, using consecutive (highly auto-correlated) monthly summaries can lead to over-performing and often unreliable models [17, 18]. Some authors have attempted to avoid these problems by using the so-called Bioclimatic Indicators or “Biovars” (e.g. maximum temperature of the warmest quarter) which are intended to represent ecological descriptors of an abiotic niche, and are assumed to suffer less from co-linearity [19].

Given that tick life-cycles are largely driven by two variables - temperature and water losses - the latter driven to a large degree by a combination of temperature and humidity [20], some authors claim that combinations of “Biovars” [i.e. 21–24] can be used as reliable covariates. Whilst an improvement on simple monthly summaries, we assert that “Biovars” are general indicators and (i) are not tailored for any species; (ii) they include interpolated rainfall data, but these are not necessarily related to the measures of humidity that are most relevant to ticks [25]; (iii) “Biovars” are simple values and at best have seasonality or timing values with a resolution of 1 month or more; and finally, (iv) “Biovars” are based on the raw monthly summaries so are also affected by co-linearity [14, 17].

A common procedure in these cases is simply to drop the affected variable(s) from the model, because they are considered “disturbing covariates”, or, in other words, variables that do not adequately train the modelling algorithms, therefore affecting its outcome. In this sense, Araújo & Guisan [26] stated that the “use of automated solutions to predictor selection … should not be seen as a substitution for preselecting sound eco-physiological predictors based on deep knowledge of the bio-geographical and ecological theory” (see also reference [27] for comments about the arbitrary selection of explanatory covariates). Studies automatically dropping covariates from a model are focused on statistical purity rather than on ecological explanations: these models will probably gain in statistical “correctness” but may lose biological relevance.

We wonder why generalist variables affected by statistical issues should be used if we can tailor our own variables for large areas and for the particular species to be modelled. Satellite - derived information has a long tradition as a descriptor of the environment affecting parasitic arthropods [i.e. 28–30].

Most remotely sensed time series of temperature and vegetation are available as 8 or 16-day composites, for periods of many years. Selection of a particular parameter as a covariate - which year, which month, which measure (mean, minimum, maximum, etc.) -  becomes something of a lottery, and the use of too many variables, e.g. weekly values, will produce over-performing models. It is therefore desirable to perform some sort of data reduction to produce relatively few variables to choose from. As there are a continuous time series of remotely sensed data, they can be reduced to their most basic temporal components, therefore reducing the redundancy (which should be avoided in any correlative modelling) while retaining ecological meaning.

One such data reduction method uses Temporal Fourier Transformations (TFT) to convert a time series of data into a mean and a number of fixed components of different periods of time (annual, biannual, triannual, etc.) each described by a phase and an amplitude. It has been reported elsewhere [31] that TFT can be used to decompose time series satellite image data into their harmonic components that are less prone to co-linearity and so more suitable to build and train correlative models. However, the harmonic regression that computes these terms also produces a series of other coefficients, which have more potential as descriptors of the climate or the vegetation seasonality, and TFT derivatives of MODIS imagery have been reported as effective and ecologically meaningful predictors for several tick species with regional or worldwide distributions [14].

A linear regression has the form y = a + bx, but in a harmonic regression the coefficients have sine and cosine transformations which capture the periodic behaviour of the values. These coefficients can be used as covariates. The regression has the form:

$$ \mathrm{y}={\mathrm{a}}_1+\left({\mathrm{a}}_2*\left(\mathrm{SIN}\left(2\pi \mathrm{t}\right)\right)\right)+\left({\mathrm{a}}_3*\left(\mathrm{C}\mathrm{O}\mathrm{S}\left(2\pi \mathrm{t}\right)\right)\right)+\left({\mathrm{a}}_4*\left(\mathrm{SIN}\left(4\pi \mathrm{t}\right)\right)\right)+\left({\mathrm{a}}_5*\left(\mathrm{C}\mathrm{O}\mathrm{S}\left(4\pi \mathrm{t}\right)\right)..\kern0.24em \right).\kern0.48em +\dots \kern0.48em \left({\mathrm{a}}_{\mathrm{n}}*\left(\mathrm{C}\mathrm{O}\mathrm{S}\left(\mathrm{x}\pi \mathrm{t}\right)\right)\right)+\left({\mathrm{a}}_{\mathrm{n}}*\left(\mathrm{SIN}\left(\mathrm{x}\pi \mathrm{t}\right)\right)\right) $$

In this regression, the “*a*’s” are the coefficients, numbered consecutively; “*t*” is the time (in days, weeks, or any chosen interval); and “*y*” is the value of the variable for that time. The first term (a_1_) is the average of the time series, while every pair of consecutive coefficients describe the slope and the duration of a seasonal change. The coefficients a_2_ and a_3_ describe the slope and the duration of spring, while a_4_ and a_5_ describe the negative slope and the duration of autumn. By replacing the values of coefficients in the equation above, the complete series can be reconstructed and can be used to reproduce the original series, which reduces the use of redundant variables [14, 32]. A script is provided in Additional file 1 that calculates these coefficients. We can obtain coefficients for as many components of this equation as necessary, but in practice it has been shown that three or four components (plus the independent term, a_1_) are sufficient for reliable correlative modelling [14, 32].

### Building models of distribution for species of ticks in the Western Palaearctic

The following examples are based on multiple logistic regression models for eight species of ticks recorded in the Western Palaearctic: *Dermacentor marginatus*, *D. reticulatus*, *Hyalomma marginatum*, *Haemaphysalis punctata*, *Ixodes ricinus*, *Rhipicephalus annulatus* and *R. bursa.* The results in the main text focus on *I. ricinus* and *H. marginatum* and are used to illustrate other features of the correlative modelling of tick distribution. The explanatory variables are the Fourier coefficients of MODIS satellite data of the daytime land surface temperature (LSTD) and the Normalized Difference Vegetation Index (NDVI), a measure of photosynthetic activity. NDVI has been used as a proxy for vegetation stress [31, 33] and can be used to derive relative humidity within the vegetation layer [31]. Both LSTD and NDVI were obtained at 1 km spatial resolution every 8 or 16 days, respectively, for the period 2001 until 2014, from the MODIS website (http://modis.gsfc.nasa.gov/data/dataprod accessed December 2014). Note that the MODIS datasets also include night-time land surface temperature, which could be used instead though this is unlikely to affect the models [34].

After calculating monthly averaged values for both LSTD and NDVI over the complete period, we obtained five coefficients of the harmonic regression for each parameter. Note that any potential correlations of the raw LSTD and NDVI [17] are removed by the calculation of TFT variables. The resulting ten explanatory variables (LSTD1 to LSTD5 and NDVI1 to NDVI5) were the only covariates included in the models. The tick occurrence dataset used in this exercise has been described elsewhere [10] and has been updated with records from the literature for this exercise, as published to December, 2014. This dataset is available as Additional file 2.

**Fig. 1:**
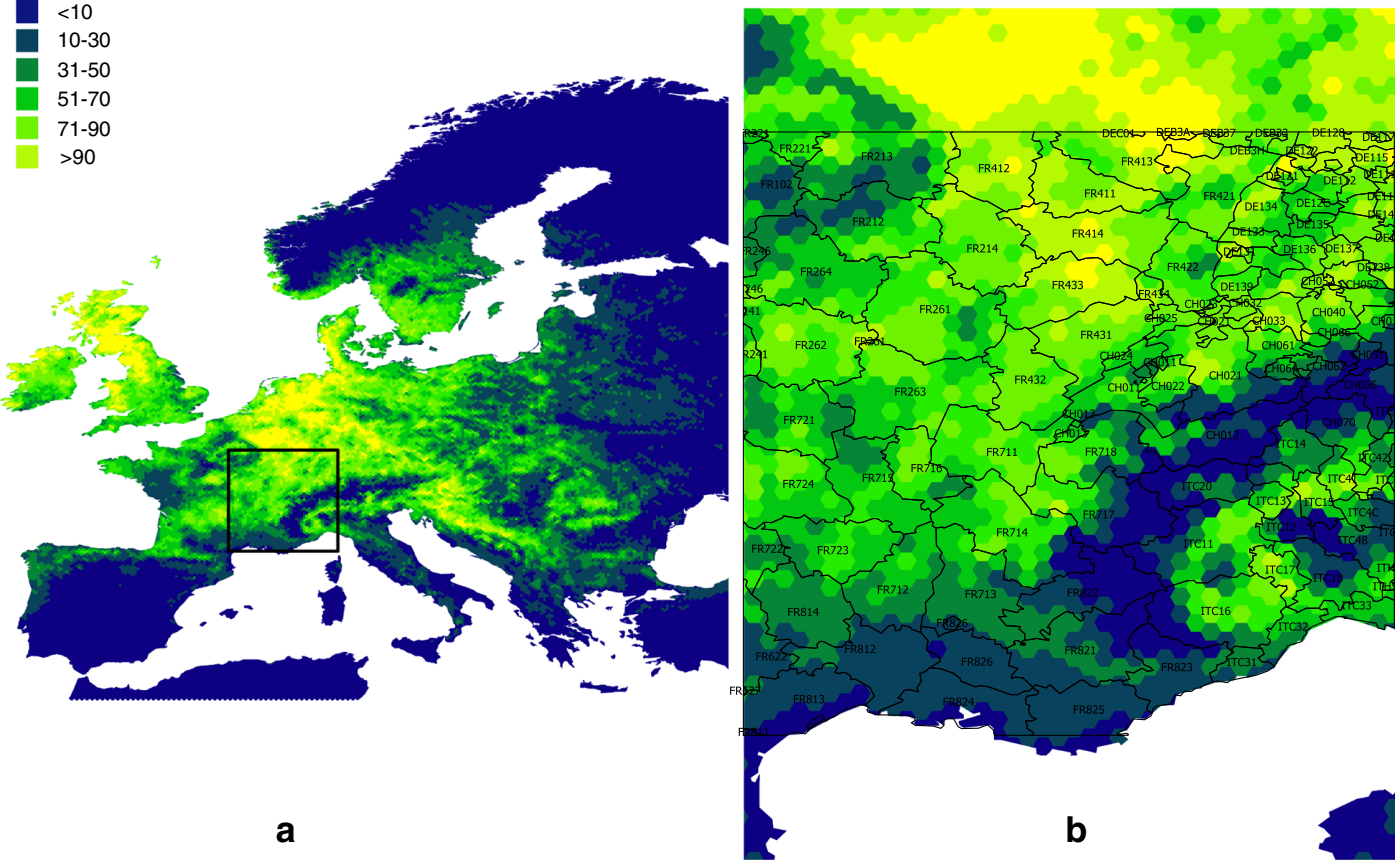
The geographical distribution of suitable environmental conditions for the tick *Ixodes ricinus* in the western Palaearctic, as obtained with the coefficients from the logistic regression shown in Table 1 and applying a grid of 0.05° to the complete target territory **a**. The method has potential not only to map such suitable conditions but to overlay with administrative divisions allowing the planning of active surveys in territories yet undetected but with positive suitability (**b**, from the square in **a**). It has also a potential for decisions makers to apply effective measurements of impact at defined territories. Because the grid covering the territory, trends of weather can also be evaluated, together with the variables shaping the distribution of a given species of tick

To reduce the impact of variability in geo-referencing of the tick dataset and to produce a common output system, we used a grid of hexagonal polygons as mapping units, at a spatial resolution of 0.1°, covering the region of interest. The use of a hexagonal grid also allows the data to be aggregated and presented for administrative divisions preferred by planners, or indeed, any other polygon areas (see Fig. 1).

The presence of each tick species was extracted for each hexagon, as was the median value of each of the ten explanatory covariates. The output model values are the probability of occurrence for each tick species converted to values ranging from 0 (unsuitable) to 100 (completely suitable). Figure 1 displays the predicted suitability for *I. ricinus.* Results for four additional species for which enough distribution points were available are provided in Additional files 3, 4, 5 and 6. Additional file 7 shows the model parameter estimates and the importance of the explanatory covariates for each of the eight models (including those where the low number of records precludes further analysis).

### Identifying the factor(s) that limit the occurrence of ticks

It has been shown [35] that the maps displaying the probability of occurrence for any organism are essentially an exercise in pattern matching and gap filling of a known distribution. They have an obvious use to show distributions, but do not explain the processes affecting the ecology of the modelled species. This limits the epidemiological conclusions that can be drawn from them, and precludes assessing the ecological consequences of changing covariates on the species’ distribution. An indication of the ecological significance of the driving variables can, however, be achieved by plotting the modelled occurrence in relation to environmental space to identify the impact of the predictors, and then looking at the geographical distributions of the predictors so identified. The former is necessary to understand the ecological determinants that affect the modelled distribution of the organism, the latter is simply the translation into a more easily visualised format. The following paragraphs provide an overview of this process.

Since ten variables were used for model building, it is desirable to reduce the dimensions of the niche to improve the readability of the resulting charts. We therefore reduced the number of dimensions by applying a Principal Components Analysis (PCA) to the results above using the R programming environment. Additional files 8 and 9 show the results of the PCA derived from the explanatory covariates. For the sake of simplicity, we show these results only for *Ixodes ricinus* and *Hyalomma marginatum* because they have widely diverging ecological requirements and their plots clearly illustrate our rationale.

The PCA outputs illustrate the contribution of the different explanatory variables to the probability of occurrence of the species. The cells with highest predicted probability of occurrence (warm colours) for *I. ricinus* (Additional file 8) have high mean values of both NDVI (NDVI1) and LSTD5, which implies small difference of temperature between autumn and winter. The picture for *H. marginatum* is different (Additional file 9), with more suitable environments implied in sites with high mean temperatures (LSTD1) and high seasonality of vegetation in both spring and autumn (NDVI2, NDVI5). Even this limited example clearly shows how a few explanatory covariates can be used to identify the factors influencing a species’ distribution, conclusions which cannot be drawn from a simple species distribution map.

### Deriving covariates with ecological meaning

The drivers identified using the PCA shown above illustrate the role of the explanatory covariates in determining the distribution of the modelled species. Whilst the reduction represented by the PCA space is both synthetic and statistically robust, its ecological meaning is sometimes difficult to capture [36]. A precise understanding of the drivers of tick distributions requires the use of ecological parameters with more widely acknowledged ecological impact, such as cumulative spring temperatures, rates of vegetation increase, or the temperature deficit below a given threshold in winter [14, 20]. These are similar to the Bioclimatic Indicators provided with interpolated datasets, but much more precisely defined to be relevant to the modelled organism.

As mentioned previously, it is possible to reconstruct the original time series on which TFT has been performed at any given temporal resolution, even days, and then build tailored variables that incorporate an ecological context into the calculated probability of occurrence. Once the daily time series is reconstructed, it is straightforward to prepare specific combinations of weather traits. A script in R is provided in Additional file 10, which imports a series of TFT coefficients derived from remotely sensed images, and computes a large set of variables that are biologically relevant for ticks [20]. The script can be easily tailored to produce other sets of derived variables, according to the organism’s requirements or assumptions used in modelling. Whilst we illustrate the use of such variables below, we emphasise there are no “silver bullets” in choosing the parameters to investigate in this way. Rather we acknowledge that the variables of significance in the climate niche may differ according to the species of tick and even the geographical context, and that selecting which to investigate is likely to be an iterative process.

These variables are not intended as potential covariates for modelling but aim to provide simple descriptions of the factors matching the predicted probability of occurrence. They are also “data driven” in that, for example, variables with a seasonal component (e.g. the sum of temperatures in spring) are based on the actual temperature changes and its slope, rather than a pre-defined date [37] and are therefore not related to the astronomical event of the change of season, which is obviously meaningless for the physiology of the modelled organisms. We encourage researchers to investigate the possibilities of producing such tailored data for any part of the Earth's surface and for any species of tick using the backbone of the script provided and using the coefficients of the TFT provided (see examples in Additional file 11).

As with the PCA plots above, these tailored covariates can be plotted against the axes of the environmental niche. Examples for *I. ricinus* and *H. marginatum* are presented in Additional files 12 and 13, respectively. The aim is illustrative, to show how different variables are associated with the predicted occurrence of ticks, and the wide range of composites with ecological meaning that can be specifically tailored for this purpose. For *I. ricinus*, we used the sum of NDVI in spring, the accumulated temperature over 0 °C in winter, the 90 % quantile of NDVI values, and the amplitude of temperature in spring. For *H. marginatum*, we used the cumulative temperature over 0 °C in autumn, the number of days over 0 °C in winter, the 75 % quantile NDVI values, and the number of days over 0 °C in autumn.

The plot shows that the predicted occurrence for *I. ricinus* follows a pattern where (i) the 90 % quantile of the NDVI is high, implying a dense vegetation layer; and (ii) the cumulative NDVI in spring is high, implying a dense vegetation layer following the winter. However, there is a weaker relation between the slope of the NDVI in spring and the predicted probability of occurrence (Additional file 12). Some of these variables may be of course correlated, but this is less relevant as our purpose is not modelling but quantifying ecologically relevant descriptions of suitable habitats.

The plot also shows that *I. ricinus* has a high probability of occurrence along a narrow range of high temperatures (measured by the values of 90 % quartile of annual temperatures and the cumulative in spring temperatures) and has a strong negative association with the slope of temperature in spring. The highest values of predicted occurrence are also where temperatures increase slowly in spring and where NDVI is high. Sites with adequate relative humidity (as measured by high values of the variables related to NDVI, which are indicators of relative humidity) are likely to support permanent populations of this tick only if cumulative winter temperature is within the defined upper and lower thresholds (measured either as the number of days above 0 °C or the cumulative temperature).

The example of *H. marginatum* identifies a different set of limiting variables (Additional file 13). High probability of occurrence is predicted where (i) vegetation is relatively poor; (ii) there are intermediate values of cumulative temperature in autumn; and (iii) there is a wide range of maximum annual temperatures. These findings are consistent with previous reports about the regulation of the life cycle of *H. marginatum* [38] in which the temperature in autumn and winter are important factors limiting the distribution of year round populations. The analysis also suggests that this species favours areas that have a comparatively low relative humidity (interpreted from the low NDVI).

We stress that this process is not *modelling* the distribution of these two species of ticks, but is an *analysis* of the ecological factors that are associated with and so may drive their modelled distribution. We do not state that the covariates identified for *I. ricinus* and *H. marginatum* are the only factors restricting its distribution, but they are illustrative of the factors governing their distributions: in this way we have added an ecological dimension to the modelling.

There are some criticisms of this approach. First, it is based on satellite imagery, which cannot measure the microclimate, which is what actually affects the development, mortality and questing activity of ticks. Secondly, it is still based on regressions, using covariates assumed to have a greater explanatory power for the life processes of the ticks. As stated at the beginning of this paper, it is well established that process-driven models would perform better [36], but we believe that if, as is generally the case, not enough is known to build area wide process based models, this type of analysis provides a viable alternative for identifying ecological drivers of tick distributions.

As seen in the previous example, the rich environmental information derived from simple datasets provides researchers with an initial look of the factors that restrict distributions or the predicted probability of occurrence. Such information cannot be extracted from a map and an algorithm processing static (and so possibly unreliable) explanatory variables.

### The use of categories of vegetation as descriptors for tick presence/absence

There have been a number of attempts to define habitat or environmental suitability for tick vectors and their hosts using land use or land cover rather than climate variables or vegetation indices. Examples focusing on ticks include (i) using vegetation categories derived from classifying satellite imagery to map the habitats of the invasive tick *Amblyomma variegatum* in the Caribbean [39] and of *H. marginatum* in the UK [40]; or (ii) the use of plant species alliances to map the distribution of *I. ricinus* for a territory in France [41, 42] or to map the reported distribution of a tick-borne virus at a national level [43].

This is a topic of potential interest, because the vegetation type has a straightforward interpretation, it can be regularly updated, is driven by climate, may reflect anthropogenic influences and change, and has the potential to describe tick habitats. Vegetation is commonly mapped at a high "resolution" (in terms of species, coverage, height, etc.) at the local or regional scale. At national or larger scale, the smaller number of predefined vegetation categories in the standard datasets may not be suitable to define tick habitats. This is simply a matter of the number of categories commonly used to draw the maps of potential vegetation, and so is not an issue of the discriminative power of the vegetation alone.

In an attempt to identify associations between tick distributions and vegetation category, we cross-tabulated the observed presence of ticks in relation to two widely acknowledged categorical descriptions of the vegetation, namely the CORINE-3 for Europe (http://www.eea.europa.eu/data-and-maps/data/corine-land-cover-2006-raster-3, accessed February 2015) and the GlobCover 2009 Scheme of Vegetation Classification (http://due.esrin.esa.int/page_globcover.php, accessed February 2015). Both of them are standardized descriptors of vegetation at continental or global scales, at a relatively high spatial resolution (around 90–300 m). We cross-tabulated the presence of each tick species and the dominant vegetation (calculated as the majority of the vegetation classes in the territory of each of the grid before). The results (Tables 1 and 2) show that whilst tick *presence* is not consistently associated with particular categories of vegetation, tick *absence* may be ascribed to a set of categorical descriptors. This suggests that, unless they are tailored to better reflect tick niches, the current schemes of vegetation categories at national or continental scales may not be effective enough descriptors of suitable tick habitats, though they could perhaps be used to describe unsuitable habitats. In addition, we believe that combining vegetation and climatic (or other) limiting factors of the sort discussed in previous sections could improve the resolution of solely climate - based environmental niche maps, which could better guide the planning of local surveys to confirm the presence of a tick species.

**Table 1:** The % tick records reported in the western Palaearctic, obtained through a systematic literature search in the western Palaearctic [15] tabulated against the CORINE-3 land cover classification scheme

Category of CORINE	*DM*	*DR*	*HM*	*HP*	*IR*	*RA*	*RB*
Agro-forestry areas	0	0	2	0	0	0	5
Annual crops associated with permanent crops	0	0	0	0	0	0	1
Broad-leaved forest	16	15	7	18	15	0	11
Complex cultivation patterns	7	11	17	6	5	30	9
Coniferous forest	3	5	2	3	17	0	1
Continuous urban fabric	2	1	6	1	1	2	4
Discontinuous urban fabric	20	22	17	17	14	13	13
Fruit trees and berry plantations	1	0	1	2	0	0	1
Green urban areas	0	1	0	0	0	0	0
Industrial or commercial units	1	4	2	0	1	1	1
Inland marshes	1	0	0	0	0	0	1
Land principally occupied by agriculture, with significant areas of natural vegetation	5	5	7	6	4	7	7
Mixed forest	2	4	2	2	10	0	1
Moors and heathland	0	1	0	1	1	0	1
Natural grasslands	1	1	4	2	1	5	5
Non-irrigated arable land	20	16	18	19	17	19	25
Olive groves	1	0	3	1	0	0	3
Pastures	10	10	2	14	9	2	2
Peat bogs	0	0	0	0	1	0	0
Permanently irrigated land	0	0	4	0	0	9	2
Rice fields	0	0	0	0	0	0	0
Sclerophyllous vegetation	1	0	1	2	0	0	2
Sparsely vegetated areas	0	0	1	1	0	9	1
Transitional woodland-shrub	3	2	4	3	3	3	3
Vineyards	2	0	1	2	0	0	1

Tabulation was done using the records in the grid against the majority of the vegetation classes in the layer of vegetation

*Abbreviations*: for the species of ticks are *DM, D. marginatus*; *DR, D. reticulatus*; *HM, H. marginatum*; *HP, H. punctata*; *IR, I. ricinus*; *RA, R. annulatus*; *RB, R. bursa*

**Table 2:** The percent of records of ticks reported in the western Palaearctic, obtained through a systematic literature search in the western Palaearctic [15] tabulated against the GlobalCov land cover classification scheme

Category of GlobalCov	*DM*	*DR*	*HM*	*HP*	*IR*	*RA*	*RB*
Artificial surfaces and associated areas (urban areas > 50 %)	6	12	17	5	5	7	9
Bare areas	0	0	1	1	0	4	0
Closed (> 40 %) broadleaved deciduous forest (> 5 m)	20	28	9	24	31	2	11
Closed (> 40 %) needle leaved evergreen forest (> 5 m)	0	2	1	1	7	1	0
Closed to open (> 15 %) (broadleaved or needleleaved, evergreen or deciduous) shrubland (< 5 m)	2	0	6	1	0	4	4
Closed to open (> 15 %) grassland or woody vegetation on regularly flooded or waterlogged soil - Fresh, brackish or saline water	0	0	0	0	0	0	0
Closed to open (> 15 %) herbaceous vegetation (grassland, savannas or lichens/mosses)	1	2	0	1	7	0	0
Closed to open (> 15 %) mixed broadleaved and needleleaved forest (> 5 m)	3	2	0	1	8	0	0
Mosaic cropland (50–70 %)/vegetation (grassland/shrubland/forest) (20–50 %)	16	5	19	16	7	14	25
Mosaic forest or shrubland (50–70 %)/grassland (20–50 %)	0	0	1	1	4	0	2
Mosaic grassland (50–70 %)/forest or shrubland (20–50 %)	1	0	0	1	1	0	0
Mosaic vegetation (grassland/shrubland/forest) (50–70 %)/cropland (20–50 %)	1	0	17	1	0	44	5
Open (15–40 %) needleleaved deciduous or evergreen forest (> 5 m)	1	1	0	0	2	0	0
Post-flooding or irrigated croplands (or aquatic)	0	0	0	0	0	1	1
Rainfed croplands	34	34	16	30	17	15	21
Sparse (< 15 %) vegetation	16	14	12	17	11	7	20

Tabulation was done using the records in the grid against the majority of the vegetation classes in the layer of vegetation

*Abbreviations*: for the species of ticks are *DM, D. marginatus*; *DR, D. reticulatus*; *HM, H. marginatum*; *HP, H. punctata*; *IR, I. ricinus*; *RA, R. annulatus*; *RB, R. bursa*

## Conclusions

Our aim has not been to compare the performance of modelling algorithms for the environmental suitability of an organism as there is already a rich literature on the topic [i.e. 44, 45]. Rather our objectives have been to illustrate how to obtain reliable estimations of environmental suitability for vectors by highlighting the importance of (i) a good set of explanatory covariates; (ii) the need to understand the ecological requirements of the target species; and (iii) deriving a set of ecologically sound information from a set of customised explanatory covariates. Our focus on ticks is especially relevant in the context of the increasing interest in (re)emerging pathogens they transmit. We have built our examples in the context of the growing tendency to simply “map” the predicted distribution of ticks using algorithms that operate on a set of interpolated datasets that do not provide adequate ecological descriptions of the observed patterns of distributions.

We suggest that the use of the widely used interpolated climatic covariates in spatial modelling of ticks can produce flawed outputs because the parameters themselves (i) may not be effective proxies for the variables that drive tick distributions; (ii) have variable accuracy depending on weather station density; and as importantly, (iii) by their very nature can be statistically inappropriate because of spatial autocorrelation and colinearity.

We have provided examples of spatial models of environmental suitability that use variables derived from harmonic regressions of remotely sensed proxies of the climatic covariates that drive distributions of ticks with widely diverging environmental constraints. Not only do these parameters have readily definable biological meaning, but they are also less prone to statistical flaws, and can produce reliable models. We have provided examples and evidence of how a gridded modelling at a continental scale is able to extract the information about the environmental suitability for ticks, at a relatively coarse scale, which can be used to design surveillance programmes or be converted to administrative level outputs for use by public health decision makers to improve preparedness and response strategies.

We have also shown that tailored covariates, created from the coefficients of the harmonic regressions, can improve upon the widely used set of bioclimatic indicators derived from interpolated datasets. The association between these variables and the modelled environmental suitability helps to identify which parameters are related to tick distributions, something that commonly cannot be achieved with pre-tailored covariates. In theory, this approach could produce a simple classification of the habitats for ticks based on remotely sensed surrogates.

We have also demonstrated that the standardized vegetation categories provided by Land use/Land cover datasets are best used to describe absence rather than presence; however, they could be used in conjunction with the climate based suitability models to enhance spatial resolutions. Such classifications (that can be obtained at other resolutions and over different regions) could then be monitored using the wide array of satellite platforms, to evaluate the environmental changes over large regions and its impact on the ecology of ticks. These thematic zones, together with an explicit evaluation of the reservoir capacity of the species of vertebrates over large regions, could eventually produce an actual estimation of the “hazard from ticks and pathogens” over wide areas.

We intend these examples to be a road map to guide researchers in producing statistically robust and biologically sound distribution models for arthropod vectors when process-driven models are unavailable, as well as using them to derive ecologically meaningful conclusions.

## Additional files

Additional file 1: A set of 6213 pairs of coordinates of ticks and hosts, as compiled from the literature. The dataset is in csv format and includes (i) specific binomial name of the tick, (ii) Name of the Genus of the tick, (iii) Specific binomial name of the host, (iv) Higher systematic details (Genus, Family and Order) of the host, (v) Country of collection, province (if not in Europe) or NUTS3 (standard denomination of European administrative units), and (vi) Latitude and Longitude in decimal degrees for 5630 records. Records that are not georeferenced records, but refer to administrative divisions, are also included for completeness. (R 2 kb) Additional file 2: A script in the R environment for programming that loads the coefficients of an harmonic regression, and calculated derived variables that can provide a more ecological meaning of the effects of climate on ticks. The example in the script calculates the derived variables for the Day Temperature, as included in Table 2 of this paper. Data calculated by this script were used to plot the Figs. 4 and 5 in the paper. (CSV 573 kb) Additional file 3: Geographic projection of the predicted probability of occurrence of *Hyalomma marginatum. (PDF 1506 kb)*
Additional file 4: Geographic projection of the predicted probability of occurrence of *Rhipicephalus bursa. (PDF 1440 kb)*
Additional file 5: Geographic projection of the predicted probability of occurrence of *Dermacentor marginatus. (PDF 1542 kb)*
Additional file 6: Geographic projection of the predicted probability of occurrence of *Rhipicephalus annulatus. (PDF 1629 kb)*
Additional file 7: Parameter estimates and metrics for the best models based on multiple logistic regression between the coefficients of Fourier harmonic regression on climate time series and seven species of ticks. There are five coefficients for the diurnal land surface temperature (LSTD) and five for the Normalized Difference Vegetation Index (NDVI). They together describe the phenology of the climate (temperature and vegetation) in the period 2001–2014. The column “Prob” displays the significance of a given coefficient in the multiple regression for each species of tick with an asterisk for those highly significant. (PDF 56 kb) Additional file 8: Principal Components Analysis decomposition of the influence of the coefficients of a Fourier regression, used to obtain the climate suitability for the tick *Ixodes ricinus*. The plot shows how the different variables used in the logistic regression are related to the modelled occurrence of the tick. In A, the predicted occurrence of the tick is plotted against the first two principal components. In B, the length and the direction of the arrows indicate how the environmental variables drive predicted tick occurrence. (PDF 742 kb) Additional file 9: Principal Components Analysis decomposition of the influence of the coefficients of a Fourier regression, used to obtain the climate suitability for the tick *Hyalomma marginatum*. The plot shows how the different variables used in the logistic regression shape the potential occurrence of the tick. In A, the predicted occurrence of the tick is plotted against the first two principal components. In B, the length and the direction of the arrows indicate how the environmental variables drive the predicted tick occurrence. (PDF 735 kb) Additional file 10: A script in the R environment for programming that loads images from MODIS repositories, transformed to monthly averages, and calculates the coefficients of an harmonic regression, as proposed in this paper as explanatory variables. The example in the script loads the monthly images of temperature for a period of several years, and saves the coefficients. (R 6 kb) Additional file 11: The list of variables derived from the coefficients of the Fourier harmonic regression, intended capture the environmental variables that restrict the distribution of the ticks. The table shows the example with temperature, and the same list of variables was calculated from NDVI. Some of these variables are auto correlated and they are not meant to develop models of distribution but should be used to understand what factors shape the predicted distribution of the focal species of tick. (PDF 24 kb) Additional file 12: Plot of the Fourier-derived variables (see Additional file 11 for a list) that partially delineate the predicted probability of occurrence for *Ixodes ricinus*. The purpose of the chart is to show the potential use of a set of traits derived from the main Fourier coefficients. The chart in A plots the quartile 90 of the NDVI in the year, and the sum of NDVI in spring. The size of the dots is proportional to the slope of NDVI in spring, and the colour indicates the predicted probability of occurrence of the tick. The chart in B plots the 90 % quartile of average annual temperature and the sum of temperature in spring, with the size of the dots being proportional to the slope of the temperature in spring. (PDF 3811 kb) Additional file 13: Plot of the Fourier-derived variables (see Additional file 11 for a list) that partially delineate the predicted probability of occurrence for *Hyalomma marginatum*. The chart in A plots the 75 % quartile of the NDVI in the year, and the annual amplitude of temperature. The size of the dots is proportional to the sum of temperature in autumn, and the colour indicates the predicted probability of occurrence of the tick. The chart in B plots the 90 % quartile of average annual temperature and the sum of temperature in spring, with the size of the dots being proportional to the slope of the temperature in spring. (PDF 2754 kb)
