# Supplementary figures and images for: Perspectives on modelling the distribution of ticks for large areas: so far so good?
Source: Parasit Vectors. 2016 Mar 31;9:179. doi: 10.1186/s13071-016-1474-9 (PMC4815247; doi:10.1186/s13071-016-1474-9)

# Additional file 3

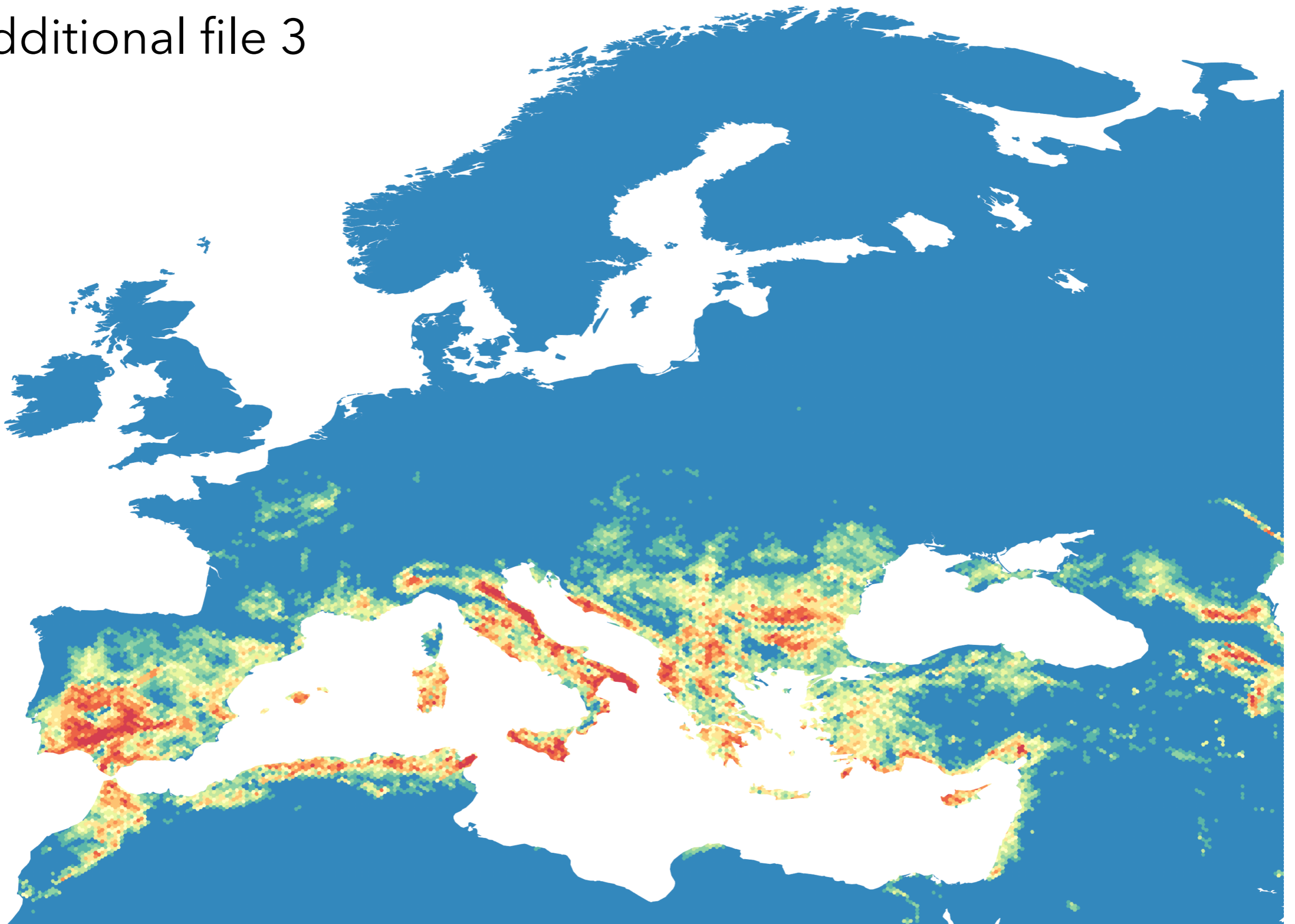

0 100

Supplement: Additional file 3: — Geographic projection of the predicted probability of occurrence of Hyalomma marginatum. (PDF 1506 kb) [file 13071_2016_1474_MOESM3_ESM.pdf]

# Additional file 4

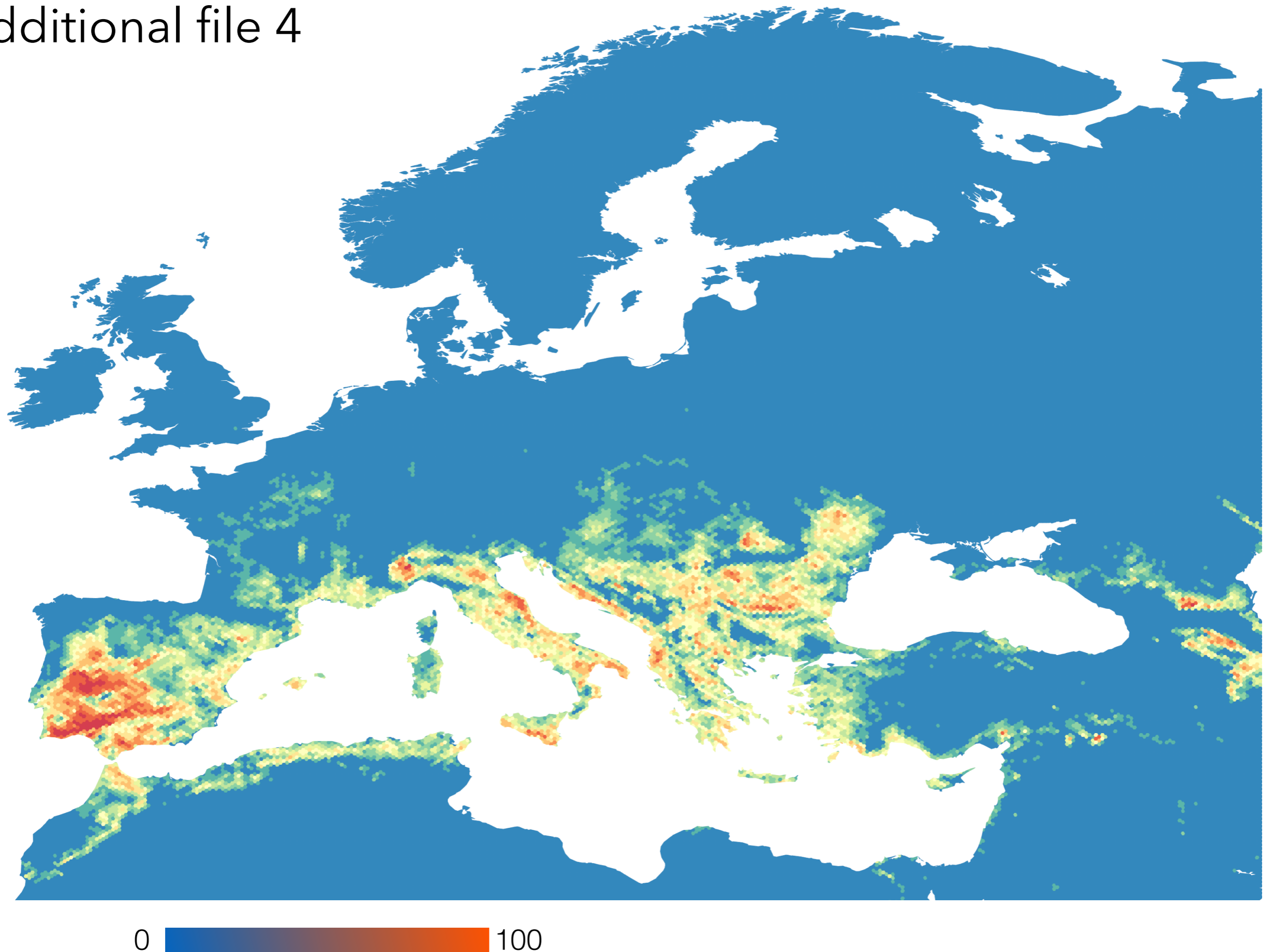

Supplement: Additional file 4: — Geographic projection of the predicted probability of occurrence of Rhipicephalus bursa. (PDF 1440 kb) [file 13071_2016_1474_MOESM4_ESM.pdf]

# Additional file 5

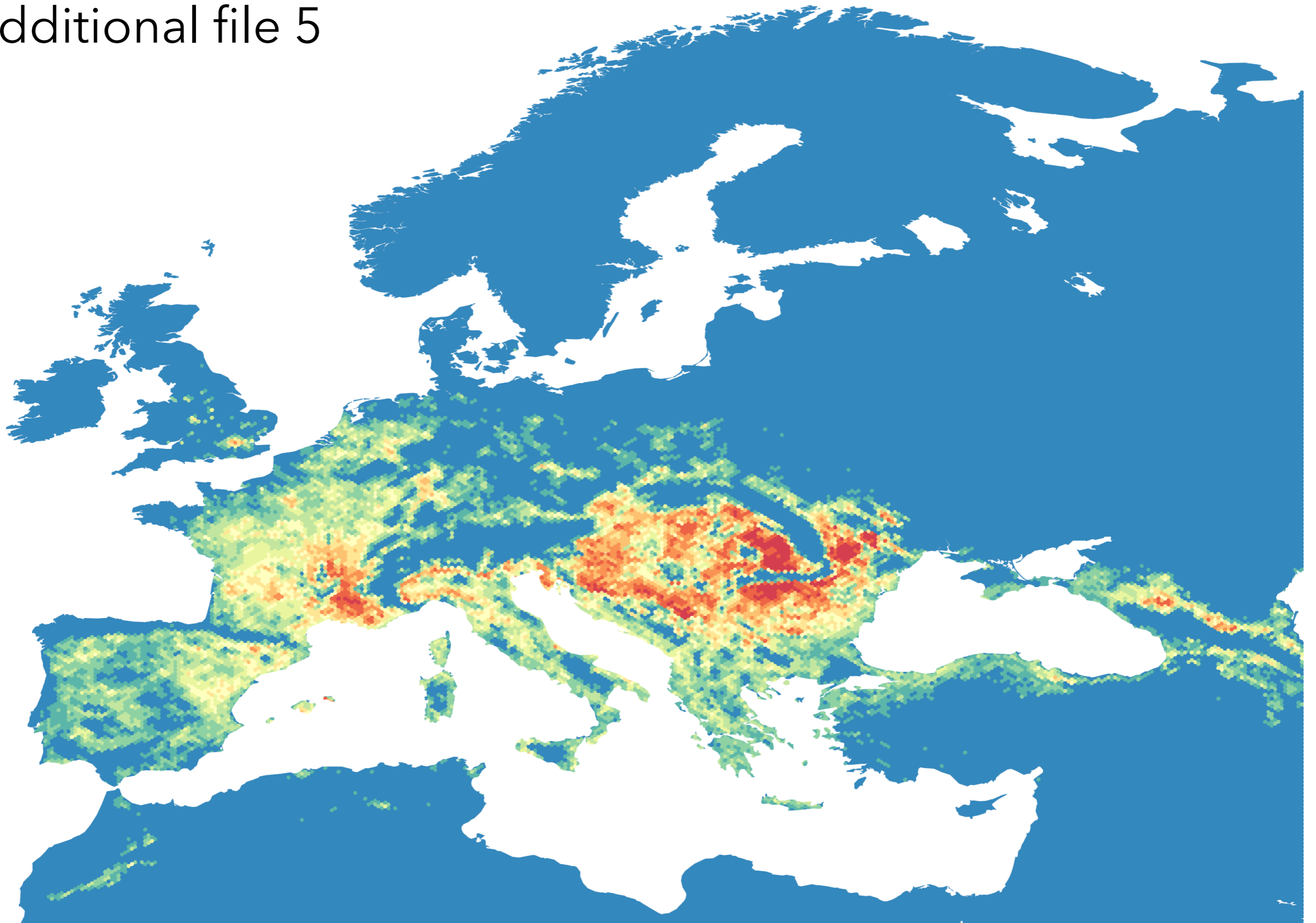

0 100

Supplement: Additional file 5: — Geographic projection of the predicted probability of occurrence of Dermacentor marginatus. (PDF 1542 kb) [file 13071_2016_1474_MOESM5_ESM.pdf]

# Additional file 6

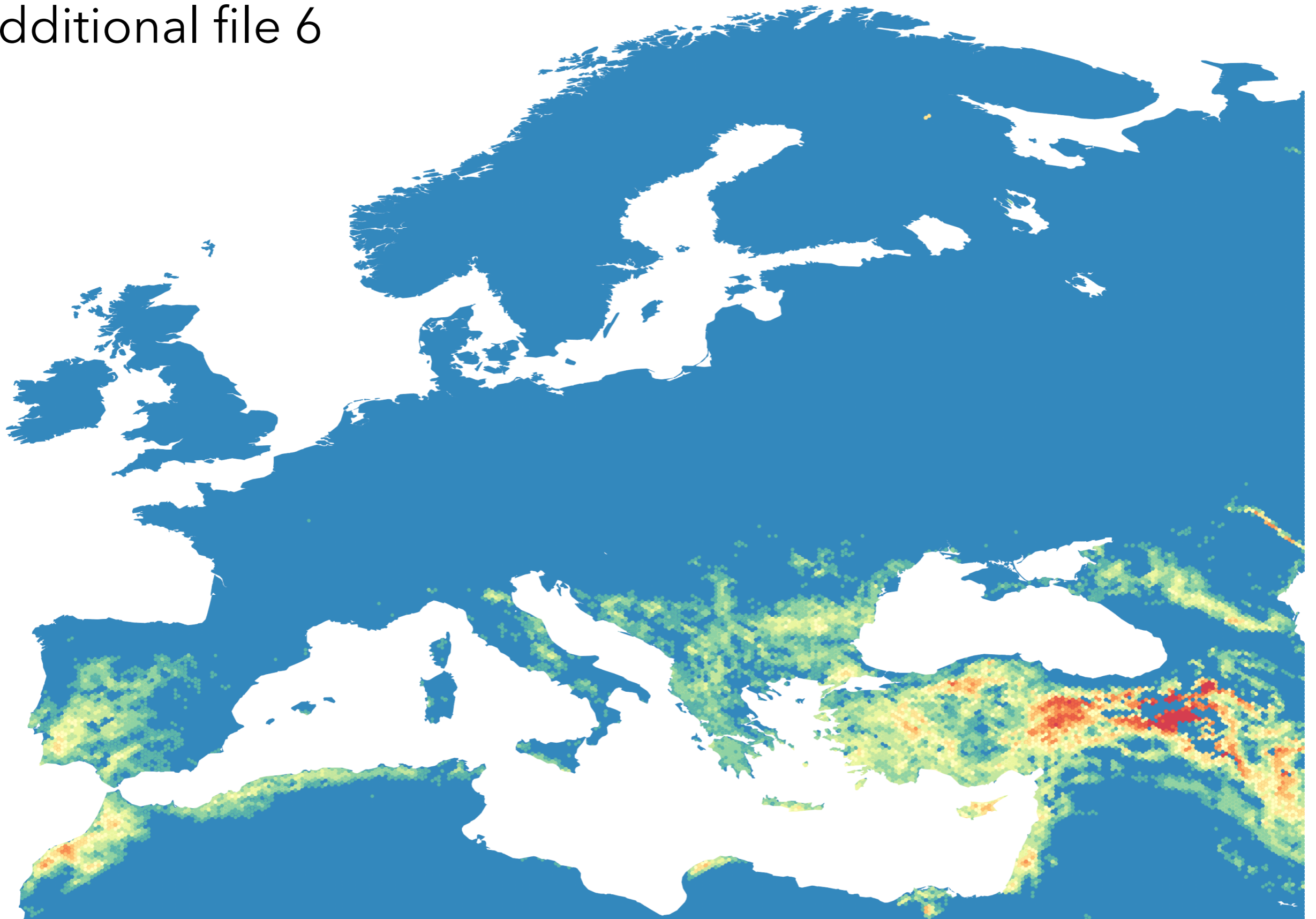

0 100

Supplement: Additional file 6: — Geographic projection of the predicted probability of occurrence of Rhipicephalus annulatus. (PDF 1629 kb) [file 13071_2016_1474_MOESM6_ESM.pdf]

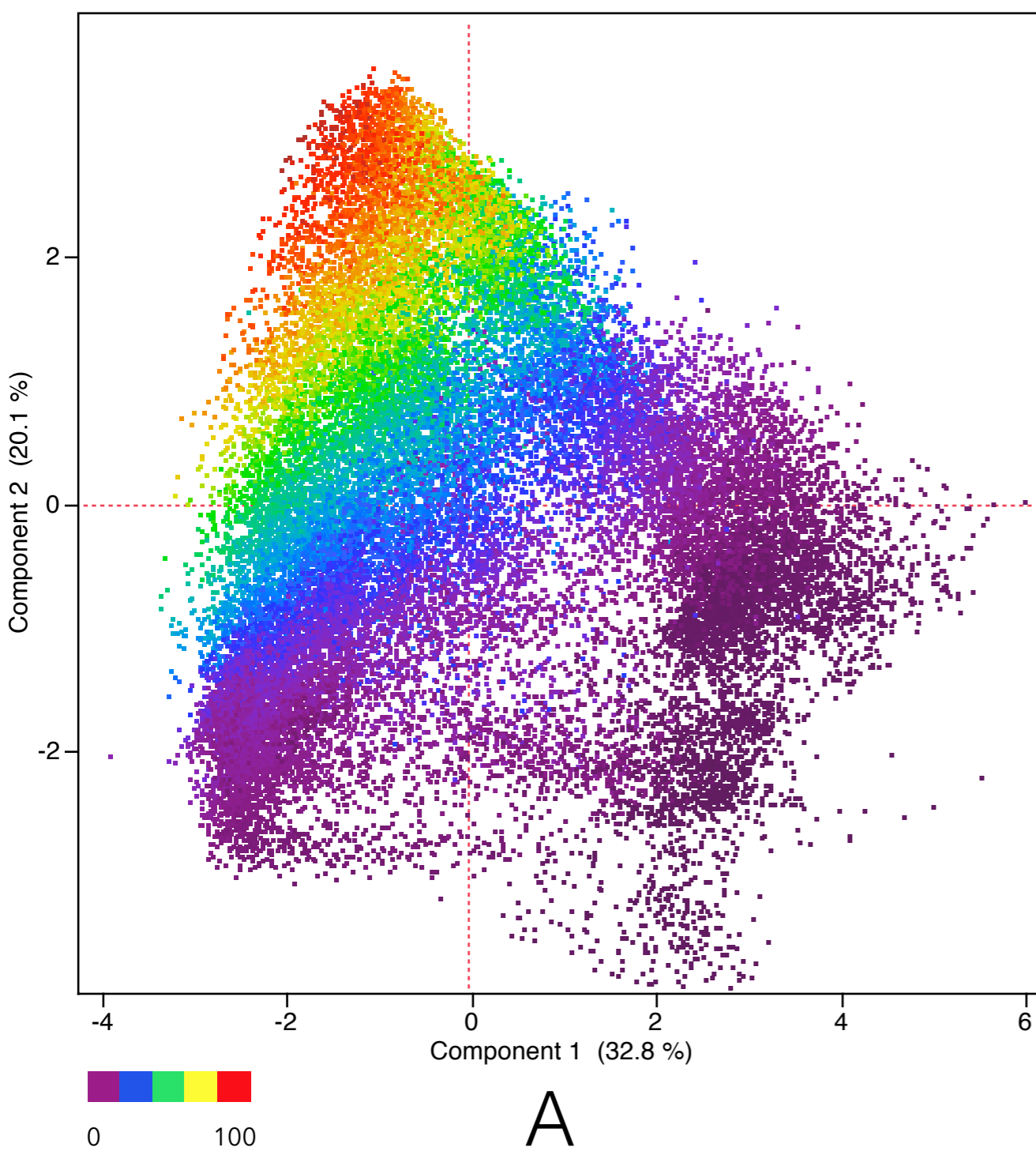

A

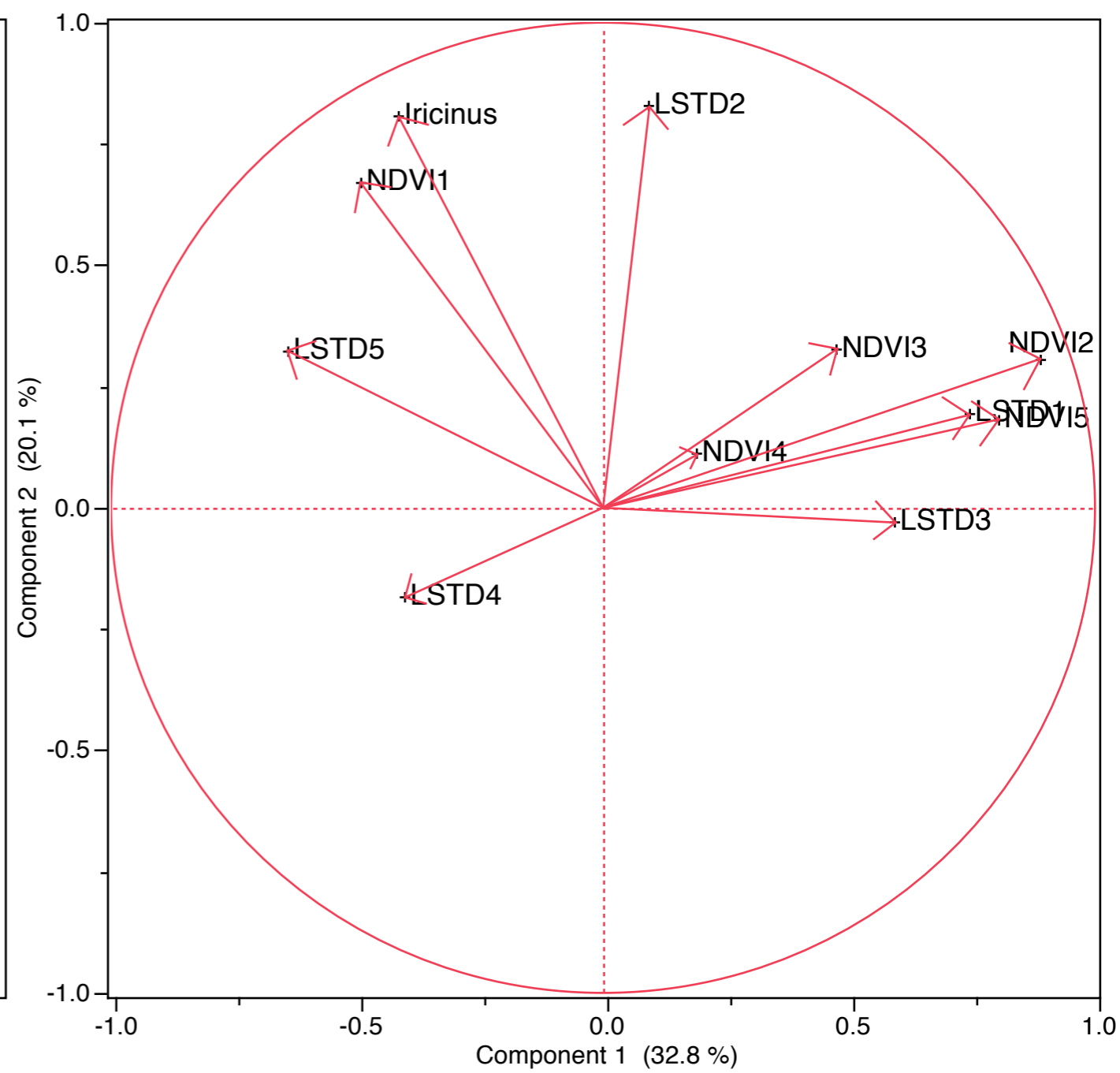

B

Additional file 8

Supplement: Additional file 8: — Principal Components Analysis decomposition of the influence of the coefficients of a Fourier regression, used to obtain the climate suitability for the tick Ixodes ricinus. The plot shows how the different variables used in the logistic regression are related to the modelled occurrence of the tick. In A, the predicted occurrence of the tick is plotted against the first two principal components. In B, the length and the direction of the arrows indicate how the environmental variables drive predicted tick occurrence. (PDF 742 kb) [file 13071_2016_1474_MOESM8_ESM.pdf]

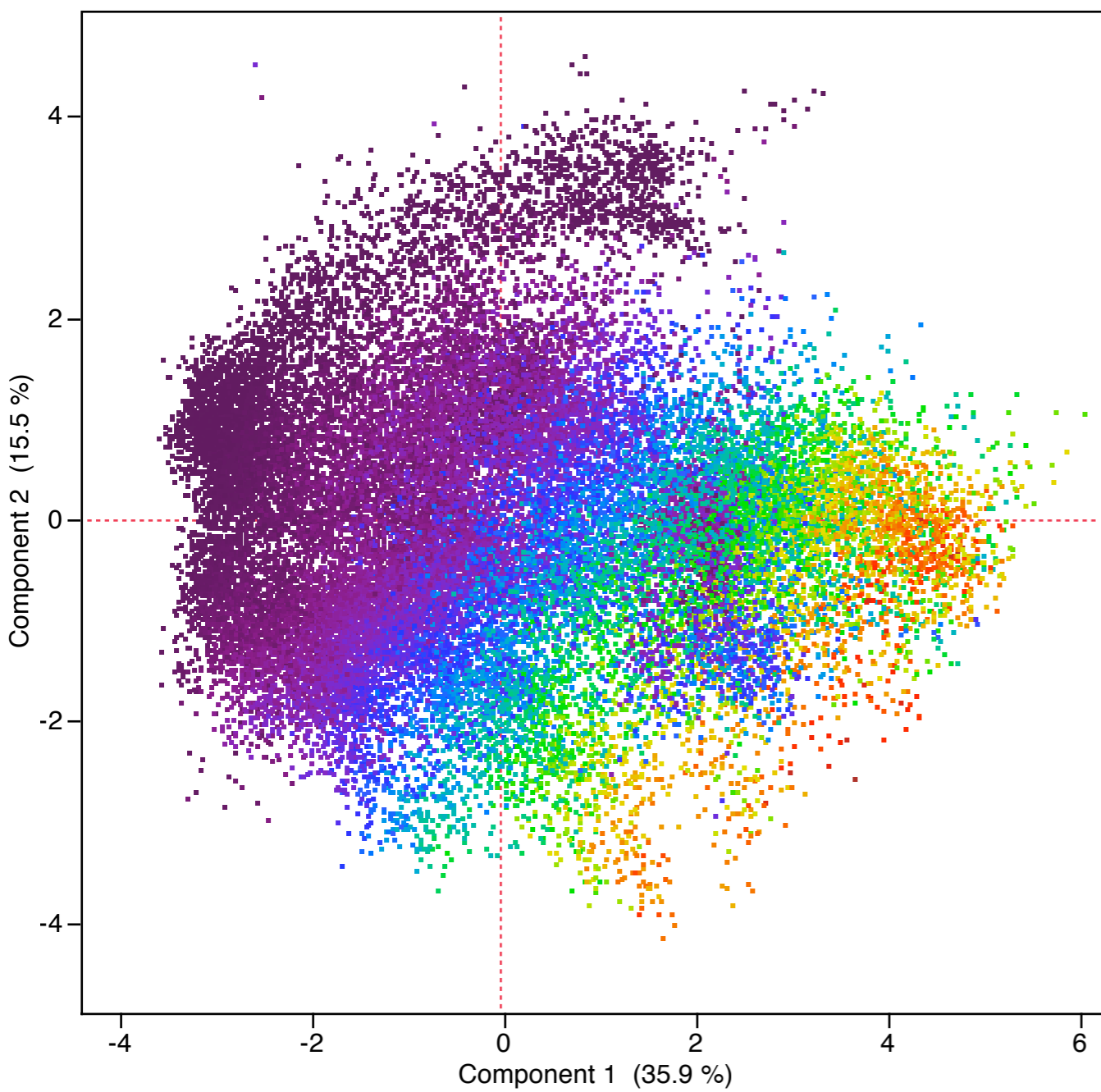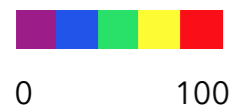

A

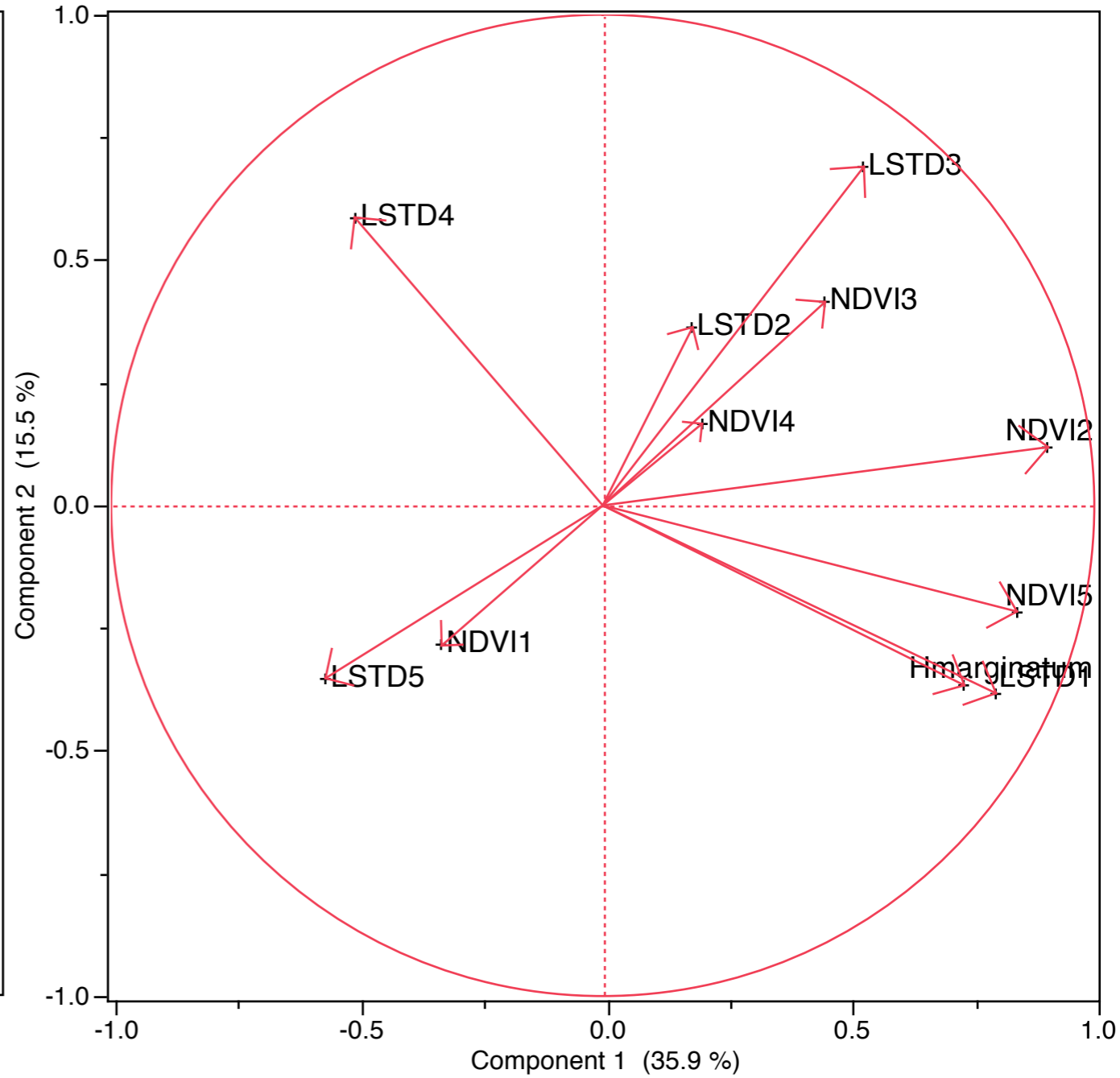

B

Additional file 9

Supplement: Additional file 9: — Principal Components Analysis decomposition of the influence of the coefficients of a Fourier regression, used to obtain the climate suitability for the tick Hyalomma marginatum. The plot shows how the different variables used in the logistic regression shape the potential occurrence of the tick. In A, the predicted occurrence of the tick is plotted against the first two principal components. In B, the length and the direction of the arrows indicate how the environmental variables drive the predicted tick occurrence. (PDF 735 kb) [file 13071_2016_1474_MOESM9_ESM.pdf]

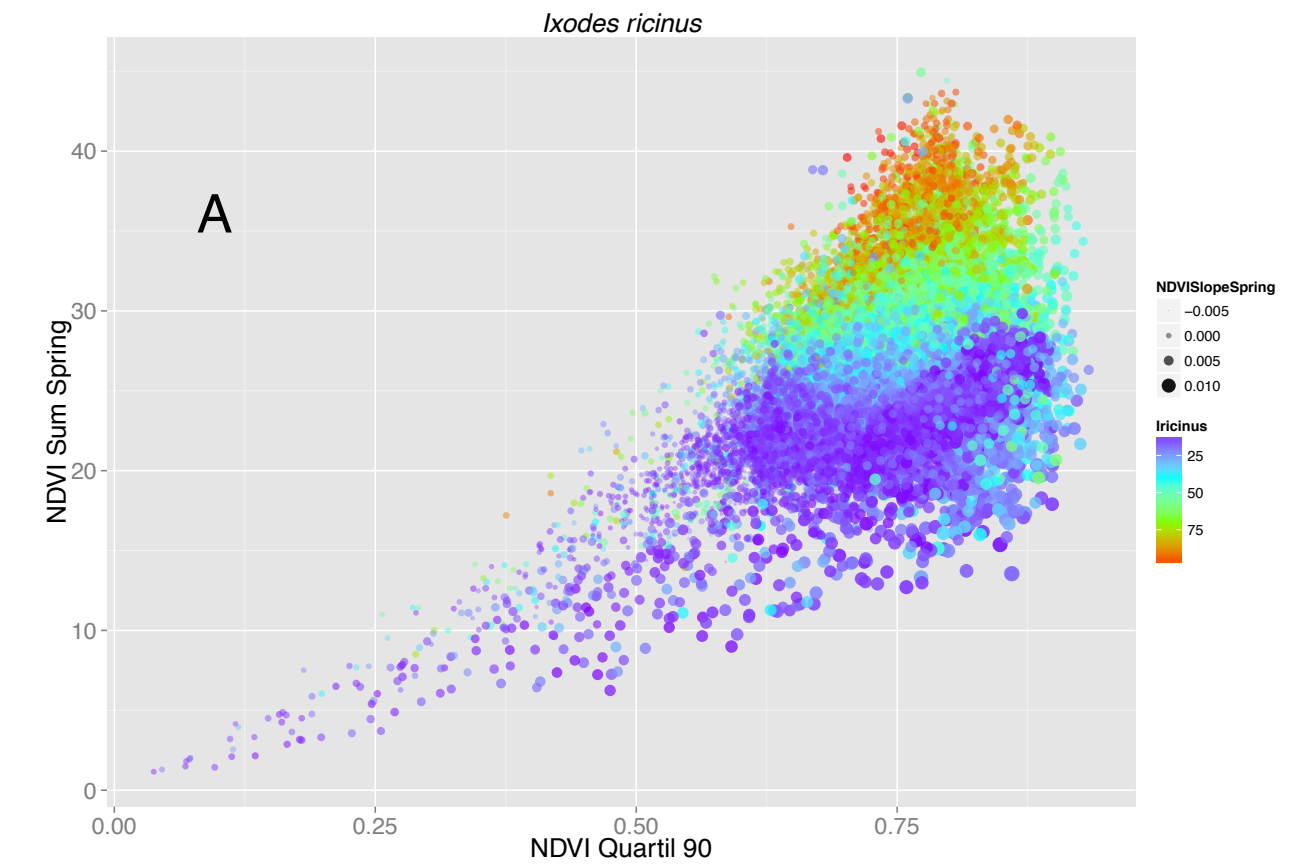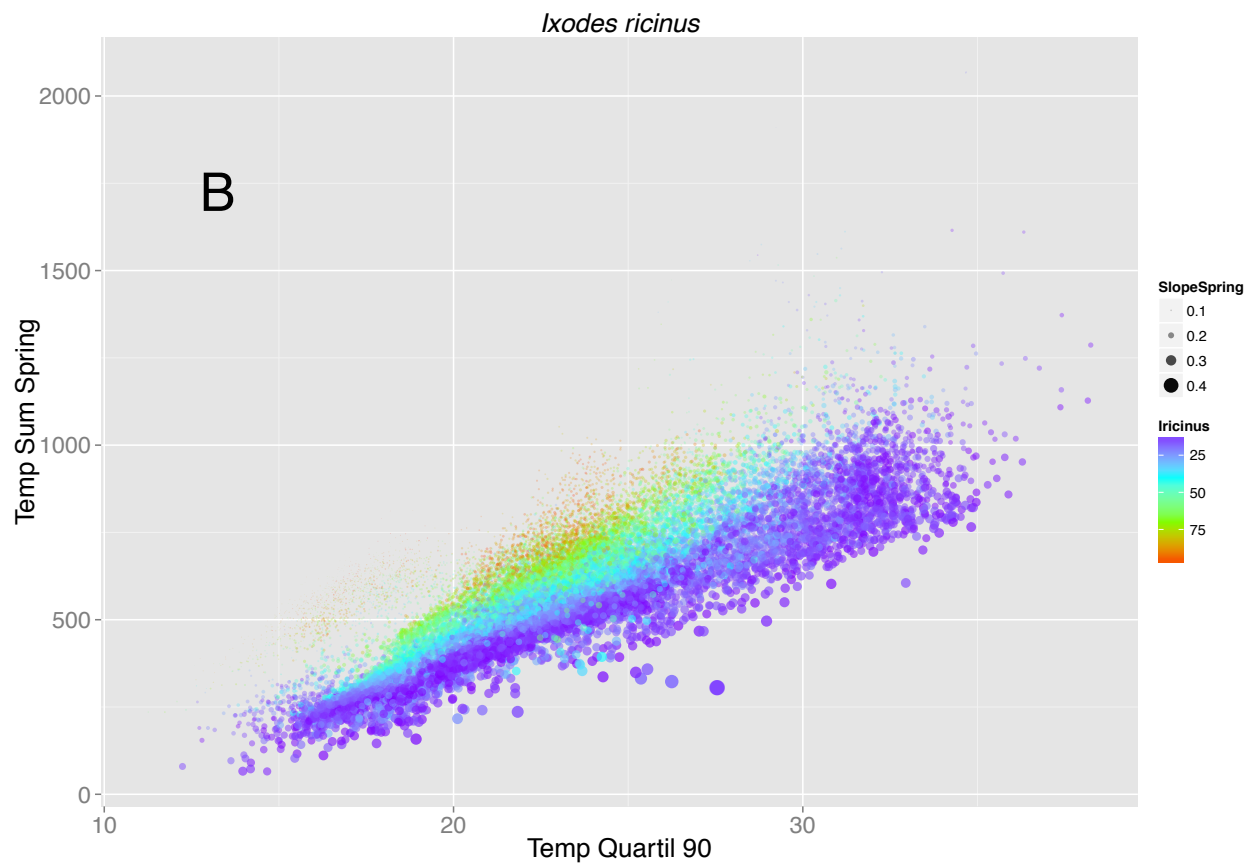

Additional file 12

Supplement: Additional file 12: — Plot of the Fourier-derived variables (see Additional file 11 for a list) that partially delineate the predicted probability of occurrence for Ixodes ricinus. The purpose of the chart is to show the potential use of a set of traits derived from the main Fourier coefficients. The chart in A plots the quartile 90 of the NDVI in the year, and the sum of NDVI in spring. The size of the dots is proportional to the slope of NDVI in spring, and the colour indicates the predicted probability of occurrence of the tick. The chart in B plots the 90 % quartile of average annual temperature and the sum of temperature in spring, with the size of the dots being proportional to the slope of the temperature in spring. (PDF 3811 kb) [file 13071_2016_1474_MOESM12_ESM.pdf]

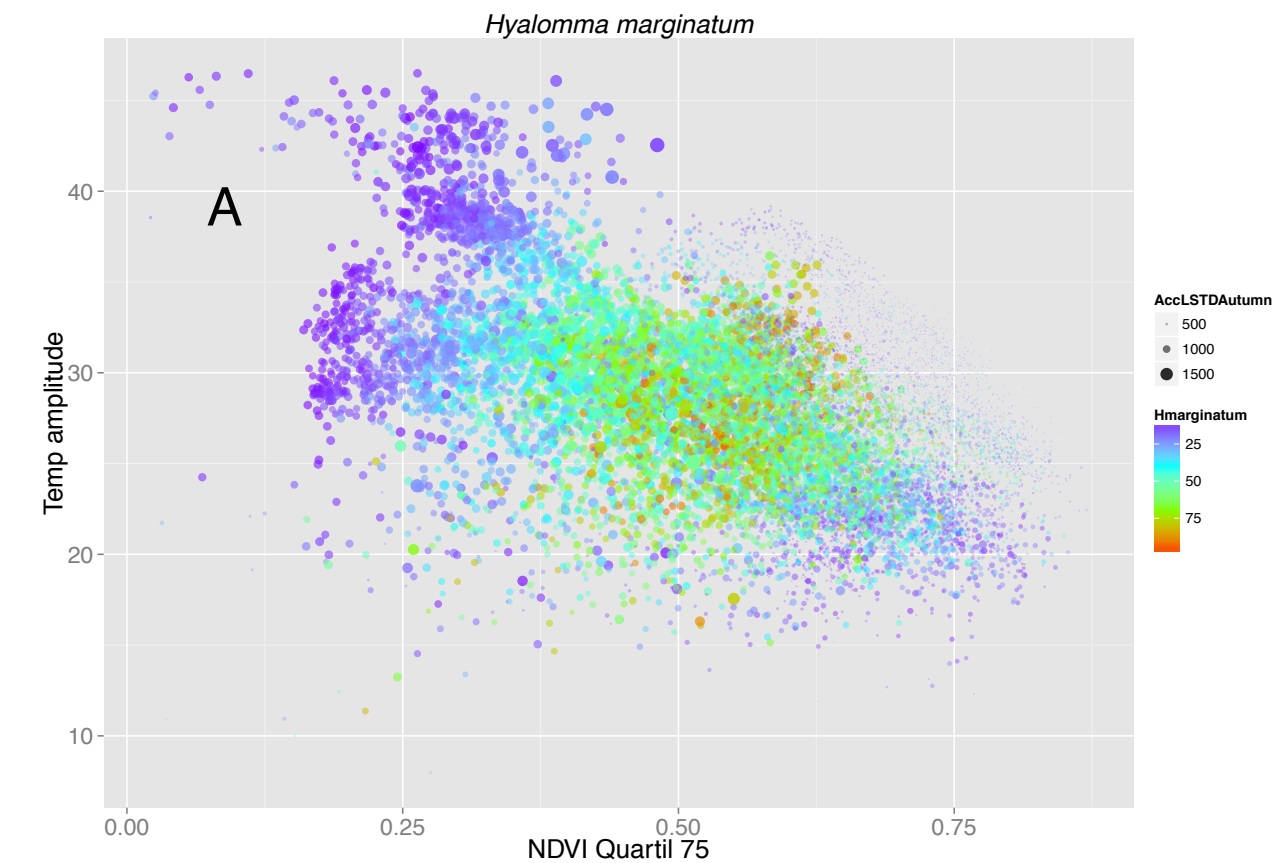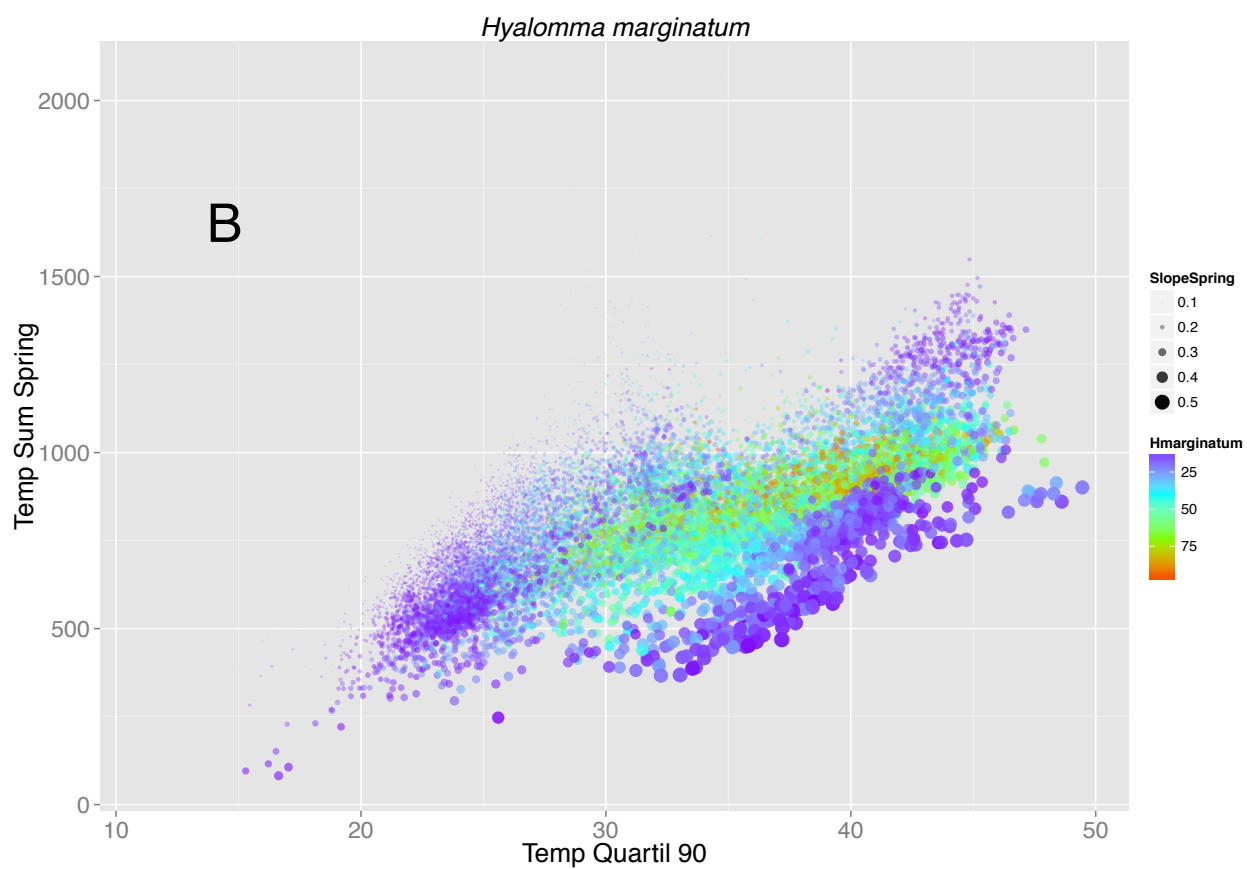

Additional file 13

Supplement: Additional file 13: — Plot of the Fourier-derived variables (see Additional file 11 for a list) that partially delineate the predicted probability of occurrence for Hyalomma marginatum. The chart in A plots the 75 % quartile of the NDVI in the year, and the annual amplitude of temperature. The size of the dots is proportional to the sum of temperature in autumn, and the colour indicates the predicted probability of occurrence of the tick. The chart in B plots the 90 % quartile of average annual temperature and the sum of temperature in spring, with the size of the dots being proportional to the slope of the temperature in spring. (PDF 2754 kb) [file 13071_2016_1474_MOESM13_ESM.pdf]
