# Supplementary material for: Perspectives on modelling the distribution of ticks for large areas: so far so good?
Source: Parasit Vectors. 2016 Mar 31;9:179. doi: 10.1186/s13071-016-1474-9 (PMC4815247; doi:10.1186/s13071-016-1474-9)
Supplement: Additional file 7: — Parameter estimates and metrics for the best models based on multiple logistic regression between the coefficients of Fourier harmonic regression on climate time series and seven species of ticks. There are five coefficients for the diurnal land surface temperature (LSTD) and five for the Normalized Difference Vegetation Index (NDVI). They together describe the phenology of the climate (temperature and vegetation) in the period 2001–2014. The column “Prob” displays the significance of a given coefficient in the multiple regression for each species of tick with an asterisk for those highly significant. (PDF 56 kb) [file 13071_2016_1474_MOESM7_ESM.pdf]

Additional file 7

	D. marginatus		*D. reticulatus*		*H. marginatum*		*H. punctata*		*I. ricinus*		*R. annulatus*		*R. bursa*	
**Term**	**Value**	**Prob**	**Value**	**Prob**	**Value**	**Prob**	**Value**	**Prob**	**Value**	**Prob**	**Value**	**Prob**	**Value**	**Prob**
Intercept	47.72	<.0001*	124.92	<.0001*	-17.55	0.0076*	21.75	0.0387*	193.76	<.0001*	-60.53	0.0005*	-23.14	0.0181*
LSTD1	-0.18	<.0001*	-0.44	<.0001*	0.05	0.0258*	-0.09	0.0094*	-0.67	<.0001*	0.18	0.0013*	0.06	0.0777
LSTD2	-0.25	<.0001*	0.02	0.7550	-0.18	<.0001*	-0.12	0.0226*	-0.10	0.0098*	-0.19	0.0042*	-0.26	<.0001*
LSTD3	-0.22	0.0522	0.62	<.0001*	-0.27	0.0012*	-0.11	0.3925	-0.30	0.0039*	-0.97	<.0001*	0.22	0.0553
LSTD4	0.98	0.0001*	0.73	0.0261*	-0.10	0.6430	-0.12	0.6811	1.73	<.0001*	-0.73	0.1268	0.29	0.2770
LSTD5	0.52	<.0001*	2.04	<.0001*	-0.43	<.0001*	0.15	0.1308	0.82	<.0001*	-1.39	<.0001*	0.02	0.8535
NDVI1	4.96	<.0001*	5.29	<.0001*	2.10	<.0001*	4.54	<.0001*	4.13	<.0001*	1.85	0.2359	4.58	<.0001*
NDVI2	0.46	0.7962	2.21	0.2926	-3.43	0.0219*	-5.62	0.0068*	5.45	0.0006*	-6.67	0.1254	-9.18	<.0001*
NDVI3	-14.79	0.0006*	-14.35	0.0062*	-2.47	0.5151	-4.10	0.4094	-13.34	0.0006*	11.79	0.1639	-0.54	0.9111
NDVI4	-0.53	0.9408	11.28	0.1785	-12.61	0.0519	-1.94	0.8110	-8.26	0.2093	-42.31	0.0010*	-24.60	0.0022*
NDVI5	-0.94	0.7227	18.31	<.0001*	1.58	0.4238	3.98	0.1878	10.23	<.0001*	-3.27	0.5294	7.95	0.0018*

Values of the best models based on multiple logistic regression between the coefficients of Fourier harmonic regression on climate time series and seven species of ticks. There are five coefficients for the diurnal land surface temperature (LSTD) and five for the Normalized Difference Vegetation Index (NDVI). They together describe the phenology of the climate (temperature and vegetation) in the period 2001-2014. The column "Prob" displays the significance of a given coefficient in the multiple regression for each species of tick with an asterisk for those highly significant
