# Supplementary material for: Perspectives on modelling the distribution of ticks for large areas: so far so good?
Source: Parasit Vectors. 2016 Mar 31;9:179. doi: 10.1186/s13071-016-1474-9 (PMC4815247; doi:10.1186/s13071-016-1474-9)
Supplement: Additional file 11: — The list of variables derived from the coefficients of the Fourier harmonic regression, intended capture the environmental variables that restrict the distribution of the ticks. The table shows the example with temperature, and the same list of variables was calculated from NDVI. Some of these variables are auto correlated and they are not meant to develop models of distribution but should be used to understand what factors shape the predicted distribution of the focal species of tick. (PDF 24 kb) [file 13071_2016_1474_MOESM11_ESM.pdf]

Derived Variable	Meaning
Interval 1	day of beginning of spring, summer, autumn or winter, respectively. It is calculated according to the slope of the series of data and not as a pure "astronomical event"
Interval 2	
Interval 3	
Interval 4	
Amplitude	Maximum minus minimum values of the variable in the complete year
Amplitude in spring	Maximum minus minimum values of the variable in each season
Amplitude in summer	
Amplitude in autumn	
Amplitude in winter	
Sum spring	The sum of the daily values of the variable in the season of reference
Sum summer	
Sum autumn	
Sum winter	
Slope spring	The angle of increase or decrease of the daily values of the variable in either spring (positive) or autumn (negative)
Slope autumn	
Quant 10, 25, 50, 75, 90	The quantiles of the distribution of daily values of the variable
Total days<0°C	The number of days in which temperature was lower or higher than 0°C
Total days>0°C	
Acc LSTD<0°C	The sum of daily values of temperature below or above 0°C
Acc LSTD>0°C	
Other calculations	The same as the previous four variables (Total days<0°C, Total days>0°C, Acc LSTD<0°C, Acc LSTD>0°C) for each season

Additional File 11
